# A Reciprocal and Dynamic Development Model for the Effects of Siblings on Children’s Theory of Mind

**DOI:** 10.3389/fpsyg.2020.554023

**Published:** 2020-10-26

**Authors:** Xiao-Hui Hou, Zhu-Qing Gong, Liu-Ji Wang, Yuan Zhou, Yanjie Su

**Affiliations:** ^1^Laboratory of Cognitive Neuroscience and Education, School of Education Science, Nanning Normal University, Nanning, China; ^2^Institute of Psychology, Chinese Academy of Sciences, Beijing, China; ^3^School of Psychological and Cognitive Sciences and Beijing Key Laboratory of Behavior and Mental Health, Peking University, Beijing, China

**Keywords:** Theory of Mind, sibling relationship, sibling interaction, preschooler, mental-state talk

## Abstract

In the field of social influences on Theory of Mind (ToM), more research has focused on the role of parents, but less research has examined the impact of siblings on children’s social understanding. We review existing research related to what factors might affect sibling–ToM association and how these potential factors affect ToM. Based on the literature review, we propose an integrative model that unites three categories of factors (i.e., sibling structural variables, sibling individual variables, parental intervening variables) that might have effects on the sibling–ToM association and highlights mental-state talks during sibling interactions at the intersection of sibling-related variables and ToM. Furthermore, we propose some issues arising from this review that need to be clarified in future studies. Specifically, we hope to clarify the specific effects of older and younger siblings on children’s understanding of human minds, the similarities and differences of sibling–ToM association under different cultural backgrounds, and the impact of family social disadvantage (e.g., lower SES) on the sibling–ToM association. All these works would benefit from the verification, revision, and expansion of our reciprocal influence model for the sibling–ToM association.

## Introduction

Theory of Mind (ToM), or mindreading, is a competence to infer one’s own and others’ mental states (such as desires, emotions, knowledge, intents, and beliefs, Wellman, 2017; Devine and Hughes, 2018). As the basis of social skills, ToM is positively correlated with prosocial behavior and peer status, while it is negatively related to antisocial behavior and peer exclusion (Watson et al., 1999; Hughes et al., 2011; O’Toole et al., 2017; Conte et al., 2018; Smorti and Ponti, 2018). It is commonly accepted that children who are developing normally acquire explicit ToM (as measured by classic false belief tasks) between the ages of 3 and 5 years, but individual differences in ToM acquisition are significant (Wellman et al., 2001). Over the past two decades, research into social influences on individual differences in ToM has primarily focused on the role of parents (Hughes and Devine, 2015; Devine and Hughes, 2018), such as parenting styles (Hughes and Ensor, 2006; O’reilly and Peterson, 2014), parental mind-mindedness (Meins et al., 2012; Mcmahon and Bernier, 2017), and parent-child talk (Ontai and Thompson, 2008; Hughes et al., 2014; Devine and Hughes, 2019). However, compared with studies on the parent–ToM association, less research has examined the impact of siblings on children’s social understanding, despite their demonstrated importance across a wide range of domains, e.g., peer relationships, social competence, and aggression (Dirks et al., 2015; Daniel et al., 2017).

A pioneering work by Dunn et al. (1991) showed that 33-month olds who engage in more cooperative interactions with their mother and older siblings do better on false belief and affective perspective-taking tasks at 40 months (Dunn et al., 1991). Such evidence has raised a series of questions: (a) Can all siblings play a crucial role in children’s understanding of mind and emotion? If no, then (b) what characteristics of siblings have an impact on children’s mindreading? And then (c) what is the specific mechanism of siblings’ influence (i.e., different from parents) on the reading of minds and emotions?

In this work, we review existing research related to what factors might affect sibling–ToM association and how these potential factors affect ToM. Note that the current review focused on explicit ToM. Implicit ToM is beyond the scope of our review. Based on the literature review, we propose an integrative model that unites three categories of factors (i.e., sibling structural variables, sibling individual variables, parental intervening variables) that might have effects on the sibling–ToM association and highlights sibling interactions (e.g., conversations, conflicts, and social pretend play) at the intersection of sibling-related variables and ToM. Furthermore, we propose some issues arising from this review that need to be clarified in future studies. Specifically, we hope to clarify the specific effects of older and younger siblings on children’s understanding of minds, the similarities and differences of sibling–ToM association under different cultural backgrounds, and the impact of social disadvantage on the sibling–ToM association. All these issues mentioned above will contribute to the verification, revision, and expansion of our reciprocal influence model for the sibling–ToM association.

## Sibling Relationships: As a Unique Context for Children’s Development

Sibling relationships are characterized by a unique combination of complementary (e.g., hierarchical) and reciprocal (e.g., egalitarian) interactions. Complementary interactions are like parent–child relationships. Sibling relationships show hierarchical characteristics due to the developmental gaps between siblings in physical (e.g., height, weight), cognitive (e.g., language and executive function), and social experiences (Campione-Barr, 2017). In the meantime, sibling reciprocal interactions are more like peer relationships. Because of the similarity in age and interests, sibling relationships exhibit the characteristics of equality, reciprocity, and sharing.

The unique nature of sibling relationships typically leads to both positive (e.g., warmth, closeness) and negative (e.g., conflict, rivalry) interactions between siblings, two indicators of the quality of sibling relationships. Positive sibling interactions (e.g., sharing, helping) are often associated with desired social outcomes. Frequent positive interactions between siblings can improve children’s ability to recognize, express, and regulate emotions; increase their prosocial behaviors; and reduce internalized and externalized problem behaviors (McHale et al., 2012; Dirks et al., 2015; Smorti and Ponti, 2018). Negative sibling interactions may give rise to either adverse or favorable effects on their social adaptation. On the one hand, frequent sibling conflicts and rivalry might increase hostile attributions to their siblings (Recchia et al., 2015) and lead to solving conflicts by aggression (Dirks et al., 2015). On the other hand, solving sibling conflicts by constructive resolutions (e.g., discussions and negotiation) provides children opportunities to understand others’ emotions and minds (Recchia and Howe, 2009). Moreover, sibling relationships show dynamic changes with age. Sibling relationships transform across time from hierarchical interactions in childhood and adolescence to egalitarian exchanges by adulthood, indicating relative power changes over time (Campione-Barr, 2017; Lindell and Campione-Barr, 2017). Regarding the characteristics mentioned above, the dominant pattern of sibling interactions may vary according to specific combinations of siblings with different characters and ages.

Sibling interactions, both complementary (e.g., teaching, caregiving) and reciprocal (e.g., joint play, conflict), offer children with ample opportunity to learn about the human mind and effective ways to interact with the social world (Dunn, 1983; Howe and Recchia, 2005; Karos et al., 2007; Harrist et al., 2014).

## Variables Related to Sibling–ToM Association

The history of sibling–ToM association studies can be roughly divided into two stages: (a) Early-stage studies have attempted to identify what aspects of sibling features can predict children’s success on ToM tasks. Early-stage studies have focused on the relationships between sibling structural variables (e.g., sibling number) or individual variables (e.g., gender) and children’s performance of ToM tasks (especially classic false belief tasks). (b) Later-stage studies have tried to explain how siblings affect children’s developing mindreading. Later-stage studies have mainly investigated the effect of sibling interactions (e.g., joint play, conflicts) on children’s mindreading. Additionally, some researchers have noticed an indirect effect of parent–child interactions on the sibling–ToM association.

### Sibling Structural Variables

Sibling structural variables refer to a series of demographic factors of the sibling composition, such as sibling number, birth order, age range, and sex composition (Lindell and Campione-Barr, 2017). Many studies have been conducted to identify what sibling structural variables might affect children’s ToM.

Initially, Perner et al. (1994) have revealed that a greater number of siblings is associated with linearly increased performance on false belief tasks in 3- to 4-year olds, and this association remained significant after age and language competence were controlled (Jenkins and Astington, 1996). However, follow-up studies have denied this positive link between sibling number and ToM ability and have instead emphasized the crucial role of birth order or age range in mindreading (Lewis et al., 1996; Ruffman et al., 1998; Farhadian et al., 2010; Calero et al., 2013; Taumoepeau and Reese, 2014). For example, some researchers have reported that birth order is a stronger predictor of ToM than sibling size (Lewis et al., 1996; Ruffman et al., 1998; Farhadian et al., 2010; Calero et al., 2013; Taumoepeau and Reese, 2014). They claimed that only older siblings can promote mental-state understanding in target children, but the presence of younger and twin siblings has no such benefits. Importantly, this positive association between ToM and the older sibling might continue into middle childhood (Cassidy et al., 2005). In contrast, other researchers have affirmed that the number of child-aged siblings (i.e., over 12 months and under 13 years of age), rather than sibling number or birth order, has positive effects on ToM. Several studies have found concurrent as well as longitudinal associations between child-aged sibling number and ToM in 3- to 6-year olds (Peterson, 2000; Mcalister and Peterson, 2006; McAlister and Peterson, 2007, 2013). Findings from special needs children (e.g., deaf or autistic children) have also supported this positive association (Woolfe et al., 2003). Furthermore, a recent meta-analysis has confirmed that the child-aged sibling number can predict superior ToM performance of preschoolers (Devine and Hughes, 2018).

In addition, only a few studies have focused on the association between sibling sex combination and ToM, and the findings are mixed. For example, according to Ruffman, children with heterosexual older siblings performed better on false belief tasks than those with homosexual older siblings (Ruffman et al., 1998). In contrast, another study has found that 4- to 11-year olds’ ability to infer others’ mental states increased with the number of same-sex siblings, even after controlling for age and executive function scores (Kennedy et al., 2015). However, a recent study has shown an interaction between older brothers and children’s sex: girls who were an only child had greater perspective-taking than girls with older brothers, whereas boys with older brothers seemed to benefit somewhat from their presence (Sang and Nelson, 2017).

Existing findings related to what sibling structural factors might affect children’s ToM development are confusing. To explain the impact of these variables on ToM, researchers have proposed an inclusive term, “sibling diversity” (Kennedy et al., 2015). They claimed that exposure to a diversity of siblings (e.g., number, sex, and age of siblings) may avail children of more opportunities to understand the differences between theirs and others’ mental states. Furthermore, the apprenticeship model (Perner et al., 1994; Ruffman et al., 1998; Hughes et al., 2014) and the age threshold model (Kennedy et al., 2015) were proposed to explain how older and child-aged siblings, respectively, can improve children’s mindreading (see section “Existing Theoretical Accounts for Sibling–ToM Relationship,” for details).

### Sibling Individual Variables

Sibling–ToM association may also be related to sibling individual variables, such as gender and personality traits. Prime et al. have found that preschoolers with an older sister rather than a brother showed advantages in mental-state understanding (Prime et al., 2016). In contrast, a study of 2-year olds from Japanese two-child families has found that the sibling’s gender did not affect their false belief understanding (Ruffman et al., 1998). Besides, some personality traits of an older sibling may impact children’s ToM through their effect on sibling interactions. For example, Prime et al. have revealed that the cognitive sensitivity (e.g., adjusting their behavior in response to their younger siblings’ knowledge level) of older siblings could predict the development of the younger sibling’s ToM after 1.65 years (Prime et al., 2016). Another study has revealed that the negative reactivity (i.e., difficult temperament) of the firstborn, measured before the second child was born, predicts sibling antagonism positively and sibling positive engagement negatively when the second child was 4–8 months old (Song and Volling, 2018). The study provided evidence for the proposal that some personality traits of siblings may impact the frequency and quality of sibling interactions and then impact children’s ToM development indirectly.

### Sibling Interaction Variables

Although a large body of studies have demonstrated that siblings benefit children’s mindreading, several studies have found that there is no difference in ToM between children with sibling(s) and only children (Downey and Condron, 2004; Lawson and Mace, 2010), and there is no relation between ToM performance and sibling number or birth order (Cole and Mitchell, 2000; Hughes and Ensor, 2005; Cutting and Dunn, 2006). These mixed findings regarding the sibling–ToM link have led researchers to turn their attention to the moderating role of sibling interactions (rather than their simple presence). It has been argued that children who are merely exposed to the social world do not extend their knowledge about the social world, but engaging in social interactions actively does (Carpendale and Lewis, 2004). At present, several process variables of sibling interaction that affect children’s ToM have been identified (e.g., conversations, conflicts, and social pretend play, Harris, 2005; Hughes and Devine, 2015).

Cooperative interactions consist of a series of positive behaviors, such as sharing, comforting, helping, timely response, and accepting suggestions. Two longitudinal studies have indicated that frequent cooperative interactions between siblings can accelerate children’s mental-state understanding. Dunn et al. (1991) have found that 33-month olds who have more cooperative behavior with older siblings did better on false belief and affective perspective-taking tasks after 7 months. Another study has found that the frequency of warmth/affection behaviors between siblings (younger *M*_*age*_ = 18 months, older *M*_*age*_ = 48 months) reported by mothers could predict the empathic concern (i.e., other-oriented emotions) level of older siblings after 18 months (Jambon et al., 2019). A cross-sectional study has found that cooperative behaviors of both a child and his/her siblings show a positive correlation with false belief reasoning (Brown et al., 1996). Further research has revealed that the frequency of both view expression and mental-state term usage increases with the cooperative level between siblings, and the frequency of mental-state terms used by second-borns is positively related to their false belief understanding (Brown et al., 1996; Hughes et al., 2006). It seems that cooperative interactions between siblings not only rely upon but also raise children’s awareness of the individual difference in mental states.

Social pretend play is another positive interaction that contributes to children’s ToM growth. It usually involves joint proposals (e.g., “Let’s play together!”), role enactment (e.g., “You pretend to be the mother, and I will pretend to be the father”), rule-making, and role-playing (Astington and Jenkins, 1995; Youngblade and Dunn, 1995; Jenkins and Astington, 2000). These activities usually give rise to conversations about desired role, clarification of play rules, free expression of ideas, and insight into the mental state of roles, all of which can enhance the mental-state understanding of the child, the siblings, and the play roles (Youngblade and Dunn, 1995; Hughes and Dunn, 1997; Howe et al., 1998). Indeed, observational studies have shown that the frequency of social pretend play and the usage of mental-state terms are two strong predictors of children’s ToM (Hughes and Ensor, 2005; Hughes et al., 2006). Besides, to ensure that social pretend play is carried out smoothly, older siblings need to be sensitive to their younger sibling’s language and social understating levels, which also deepens the social understanding of children (Prime et al., 2016; Derksen et al., 2018).

Sibling conflict is the third way of contributing to children’s understanding of others’ emotions and minds. Whether sibling conflict can promote ToM ability may be moderated by the quality of sibling relationships and conflict-solving strategy (Slomkowski and Dunn, 1992; Foote and Holmes-Lonergan, 2003). For example, high-quality sibling relationships increase the tendency for constructive conflict resolutions (e.g., negotiation) and reduce destructive conflict resolutions (e.g., aggression; Recchia and Howe, 2009). Furthermore, constructive conflict resolutions, which need siblings to take account of one another’s feelings and express their viewpoints, may also stimulate awareness of contrasts in desires, emotions, and intentions (Hughes et al., 2006; Ram and Ross, 2008; Recchia and Howe, 2009). Indeed, facing sibling conflicts, 3- to 5-year olds who adopted other-oriented debates (e.g., referring to siblings’ intentions and feelings) used more mental-state terms and did better on false belief tasks relative to children who adopted self-oriented debates (i.e., expressing their desires, feelings, and ideas only) or did not engage in debates (e.g., verbal or physical attacks, Foote and Holmes-Lonergan, 2003). These findings indicate that conflicts have often provoked sibling conversations (e.g., talking about emotions and causality) that can be conducive to the understanding of others’ emotions and minds (Kristin and Wellman, 2002).

### Parents’ Involvement in Sibling Interactions

In long-term and intensive interactions (e.g., family conversations, social pretend play, and arguments), siblings perceive, infer, and talk about the mental states of themselves and others, and therefore, their understanding of the causality between mental states and behaviors is constantly deepened (Howe et al., 2011; Leblanc et al., 2017). At the same time, interaction with parents might moderate the association between the sibling and ToM by affecting the quality and quantity of sibling interactions.

Parents play a key role in nurturing a positive sibling relationship and reducing or even eliminating the negative effect of sibling rivalry/conflict. Therefore, parent–child interactions might moderate the association between siblings and ToM by affecting the quality and quantity of sibling interactions. For example, if mothers emphasize that firstborns have the responsibility to take care of newborns, firstborns benefit from building positive sibling relationships (Dunn and Kendrick, 1982), and this caretaking level can predict later positive sibling interactions (Song and Volling, 2015). More importantly, parent–child conversations focused on newborns may enhance earlier-borns’ appropriate responses to the emotions and needs of newborns (e.g., comfort, caretaking; Slomkowski and Dunn, 1992; Randell and Peterson, 2009).

Parents may also intervene in sibling conflicts, which often provoke mental-state talk (e.g., talking about the causes of negative emotions) between a parent and a child (Kristin and Wellman, 2002), which can be conducive to the development of ToM (Lagattuta and Wellman, 2002). In a further extension, several studies have documented that parental discipline moderates the association between sibling conflict and mindreading. Indeed, if parents frequently adopted parent-centered disciplines (e.g., scolding or threatening) or rarely adopted child-centered disciplines (e.g., negotiation), children were unlikely to benefit from sibling conflicts (Song and Volling, 2018). By focusing on the mental states of both sides, child-centered disciplines provided children with opportunities to intuitively understand others’ needs, emotions, and beliefs (Hughes et al., 2011). Parent-centered disciplines may inhibit children’s emotional regulation and mindreading by adopting punitive rather than mentalistic conversations (Dunn and Kendrick, 1982; Perozynski and Kramer, 1999). In the first year of life for a newborn, the more punitive the methods that parents use, the more likely that sibling relationships will fall into the early onset antagonism class, which might weaken or eliminate the association between the sibling and ToM (Oh et al., 2015).

In sum, there are a variety of processes (both direct and indirect, and positive and negative) that are likely to mediate or moderate the “sibling effects” on individual differences in ToM ability.

## Existing Theoretical Accounts for the Sibling–ToM Relationship

To explain how older siblings and child-aged siblings contribute to ToM development, researchers have proposed two models, namely, the “apprenticeship model” and the “age threshold model.” In Table 1, we summarize the differences between the apprenticeship model and the age threshold model in four aspects, including key points, key variables, influence mode, and related theory.

**TABLE 1:** Comparison of the apprenticeship model, the age threshold model, and the reciprocal and dynamic development model.

	Apprenticeship model	Age threshold model	Reciprocal and dynamic development model
Key points	(a) Older siblings can unilaterally promote younger sibling’s ToM development (b) This “sibling effect” positive	(a) Interaction between child-aged siblings accelerate each other’s ToM growth (b) This “sibling effect” positive	(a) Sibling interaction promote each other’s mental state understanding (b) This “sibling effect” either positive or negative (c) This sibling-ToM association is dynamic developing with age
Key variables	(a) Birth order (b) Sibling complementary interaction	(a) Child-aged sibling (b) Sibling reciprocal interaction	(a) Age of sibling dyads (b) Quantity and quality of mental-state talks
Influence mode	Older siblings provide younger children with teaching, guidance, and scaffolding	Social pretend play, cooperation, and conflicts between siblings	Mental-state talks between parents and children
Related theory	Vygotsky’s sociocultural theory	Piaget’s theory of cognitive development	(a) Vygotsky’s sociocultural theory; (b) Piaget’s theory of cognitive development

### Apprenticeship Model

As shown in Table 1, the apprenticeship model tries to explain why children with older rather than younger/same-age siblings show superiority in mindreading. (a) The model holds that preschoolers with older siblings demonstrate superior ToM performance relative to children with younger or twin siblings. (b) The model holds that the presence of a sibling provides children with plenty of social opportunities that improve their knowledge about human mental states, with older siblings being superior because they play a social mentor role to the younger “apprentices” (Hughes et al., 2014; Kennedy et al., 2015). (c) The model emphasizes the unidirectional effect of older siblings on younger children (Perner et al., 1994; Ruffman et al., 1998). Indeed, younger children, due to their limited receptive and/or expressive language and lack of social experiences, are unable to benefit older siblings (Brown et al., 1996). (d) The apprenticeship model is rooted in Vygotsky’s cultural–historical theory of psychological development, which asserts the unilateral influence of the increased amount of knowledge of the other (e.g., parents, teachers, and older siblings) on developing individuals (Vygotsky, 1978).

### Age Threshold Model

As illustrated in Table 1, the age threshold model attempts to explain why child-aged siblings rather than an infant or adolescent sibling can enhance the understanding of minds. The core points of the age threshold model are as follows (Kennedy et al., 2015): (a) Children who have one or more child-aged siblings outperform only children in ToM tasks, and the presence of an infant or teenager and above exerts no benefit. (b) Siblings afford children frequent exposure to and participation in social life that is related to social-cognitive growth (Peterson, 2000; Dunn, 2015). (c) The age threshold model emphasizes the effect of reciprocal interactions between child-aged siblings on social understanding. When younger children reach a certain age, where both sibling dyads are qualified with linguistic ability and social experiences that enable them to develop effective interactions (e.g., joint play, arguments), both can promote each other’s mental-state understanding. (d) The age threshold model can be traced back to Piaget’s theory of cognitive development, emphasizing that peer interactions (especially conflicts) play a crucial role in social-cognitive development.

### Summary

The apprenticeship model provides reasonable explanations for the association between older siblings and ToM of 3- to 6-year olds, when the sibling relationships mainly manifest as complementary interactions (Karos et al., 2007; Harrist et al., 2014). However, the age threshold model suggests that having a child-aged sibling at home is a stronger predictor of mindreading than the number of older siblings (Peterson, 2000; Dunn, 2015; Kennedy et al., 2015). These two models provide reasonable explanations for the “positive” effect of older or child-aged siblings on children’s social understanding, respectively. However, two recent studies have found that infant or toddler siblings harm preschoolers’ ToM (see section “Summary” for details; Leblanc et al., 2017; Paine et al., 2018). We speculate that there might be different mechanisms between younger/same-age and older siblings in promoting target children’s mental-state understanding. Therefore, a more integrative model is proposed to organize these mixed findings in the next section.

## A Reciprocal and Dynamic Development Model for Sibling–ToM Association

**FIGURE 1:**
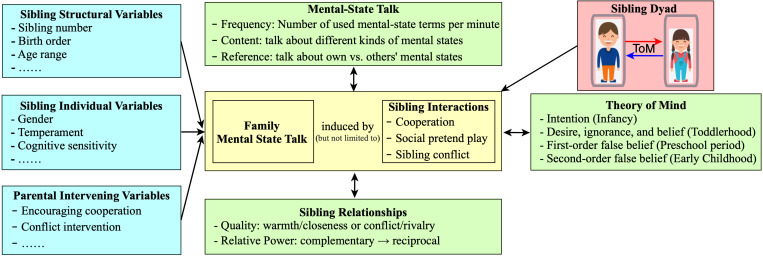
A reciprocal and dynamic development model for sibling–Theory of Mind (ToM) association. Three kinds of sibling–related variables that might affect children’s ToM have been identified (cyan). The mechanism underlying the sibling–ToM link is that having siblings leads to more opportunities for children to be exposed to and take part in family mental-state talk among parents, siblings, and target children, providing unique insights into the workings of the social world. Here, it is worth noting that family mental-state talk could be induced by but not limited to sibling interactions. For example, mental-state talk is also induced by parent–child reading (yellow). On the other hand, this model underlines the reciprocal and divergent influences of older and younger siblings. Specifically, we suspect that older siblings might continuously promote younger children’s ToM development from toddlerhood to childhood of the later-born child. By contrast, there might be an inflection point, where the direction of younger sibling–ToM association transforms from negative (during the first year of the later-born child’s life, parents need to spend more time and effort on infant or toddler caretaking, which poses an obstacle to the development of the ToM ability of older siblings) to positive, when the later-born child is growing into a more skilled playmate from the second year of his/her life (red). On the other hand, given that children’s ToM, the nature of the family mental-state talk, and sibling relationships are all constantly changing with age, this model claims that the sibling–ToM link is a dynamic process but not a static concept (green). This model highlights the interrelations among relationship quality, interactions, and ToM. Specifically, the quality of sibling relationships influences the frequency and quality of sibling interactions; frequent and intense interactions between siblings can shape the development of mindreading abilities and sibling relationships.

By integrating existing literature and unresolved problems, the current review puts forward an integrative theoretical model for the sibling–ToM association (see Figure 1 and Table 1). This model describes what factors might affect the sibling–ToM link and then explains how these potential factors affect ToM. Upon reviewing the three categories of factors that might have effects on the sibling–ToM association, a picture emerges of the mental-state talks during sibling interactions at the intersection of sibling-related variables and ToM. Moreover, this model emphasizes the reciprocal and dynamic development of the sibling–ToM association.

### The Underlying Influence Factors of Sibling–ToM Associations

It is revealed from our model, as illustrated in Figure 1, that the association between the sibling and ToM is affected by multiple factors. Furthermore, these factors can be classified into three categories: (a) sibling structural variables, including sibling number, birth order, and age range; (b) sibling individual variables, e.g., gender, temperament, and cognitive sensitivity of the sibling; and (c) parental intervening variables, e.g., encouraging cooperation and conflict intervention. Our model positions mental-state talks during sibling interactions at the center of the three categories of factors and ToM. Three kinds of factors play a role in ToM growth by acting on the quality and quantity of sibling interactions, which often induce mentalistic conversations between the parents and children or between the sibling and the target child (e.g., discussing the reason for sibling disputes, negative emotions). Therefore, the mechanism underlying the “sibling effects” of ToM is that the presence of siblings leads to more opportunities for children to be exposed to and take part in mental-state conversation among parents, siblings, and target children.

### Several Features of Mental-State Talk That Mediate Sibling–ToM Association

Numerous studies have been conducted to identify what aspects of mental-state talk foster children’s mindreading. Several features of mental-state talk, including frequency (i.e., number of mental-state terms mentioned per minute), category (i.e., talk about different kinds of mental states), and reference (i.e., talk about own vs. others’ mental states) have been identified that mediate the sibling–ToM association (De Rosnay and Hughes, 2006; Tompkins et al., 2018). The results from a study in 2-year olds and their old siblings indicate that later-borns’ performance on ToM tasks is associated with both frequency and variety of the sibling mental-state talk during reciprocal/pretend play (Hughes et al., 2006). Another study has revealed that 4-year olds talk more about their own rather than their siblings’ mental states during play at home, but there is a significant association between a reference to their siblings’ emotion/desire and ToM performance (Hughes et al., 2007). Moreover, other- rather than self-oriented arguments adopted by preschoolers in sibling disputes are positively correlated with their scores on false belief tasks (Foote and Holmes-Lonergan, 2003). In contrast, when social partners change from siblings to parents or friends, cognitive state talk is a stronger predictor of false belief and emotion understanding relative to desire/emotion talk (Hughes et al., 2007; Tompkins et al., 2018). These data indicate that even if children more frequently talk about their own mental states (as revealed by Hughes et al., 2007), the references to siblings’ mental states matter and benefit their ToM skills, during both positive (e.g., social pretend play) and negative (e.g., conflict) interactions with their siblings.

### The Reciprocal and Divergent Influences of Older and Younger Siblings

The apprenticeship model asserts that older siblings unilaterally promote younger children’s ToM development. In contrast, the age threshold model claims that interactions between child-aged siblings accelerate each other’s ToM growth. These data indicate that previous studies have not reached a consensus about sibling influences on ToM that is unidirectional or reciprocal. Two recent studies have even found that younger siblings (e.g., infant or toddler) could be an obstacle to the mindreading development of the firstborn child, especially when the sibling age gap is less than 2 years (Leblanc et al., 2017; Paine et al., 2018). One possibility for this result is that parents need to spend more time and effort on infant or toddler caretaking, which poses an obstacle to developing the ToM ability of older siblings (Baydar et al., 1997; Wright and Mahfoud, 2012). Indeed, studies on the two-child family have shown that firstborns show superior ToM performance only when the second-born child is growing into a more skilled playmate (Lagattuta et al., 2015; Leblanc et al., 2017; Paine et al., 2018). Therefore, we speculate that sibling influences on ToM are reciprocal, and the direction of this “sibling effect” can be either negative or positive.

Moreover, we suspect that older and younger sibling–ToM associations would show divergent developmental trajectories. Specifically, from the second year of late-borns, older siblings might begin to improve their ToM by active interactions between siblings and exposure to parent–older sibling mental-state talk. In contrast, later-borns under the age of 1 might impede their older siblings’ ToM ability. In the second year of life of a later-born, he/she begins to actively engage with siblings (Dunn, 2014; Jambon et al., 2019); thus, sibling interactions allow them to provide one another with a potent context to learn about effective ways to interact with the social world.

### Interrelations Among Relationship Quality, Interactions, and ToM

Here, it is noted that the quality of sibling relationships, sibling interactions, and ToM are interconnected. Relationships between siblings differ greatly in quality; only those sibling dyads who are higher in closeness, warmth, and affection engage in frequent cooperative interactions and play, and resolve a conflict by negotiation (Howe et al., 1998; Howe et al., 2002; Cutting and Dunn, 2006; Recchia and Howe, 2009). In turn, frequently positive interactions (e.g., caring) at home will certainly foster intimate, warm, and affectionate relationships between siblings (Dunn and Kendrick, 1982; Song and Volling, 2015).

Similarly, the association between sibling interactions and ToM is also believed to be bidirectional. On the one hand, ToM facilitates sibling interactions, such that children with a high level of mindreading skills establish and maintain smooth play and conversations. On the other hand, sibling interactions can serve as a type of training to improve their social cognition and social skills. Several longitudinal studies have reported that positive (or negative) sibling interactions at an early stage can promote (or hinder) children’s mindreading at a later stage (Dunn and Kendrick, 1982; Song et al., 2016; Song and Volling, 2018). Also, constructively resolving conflicts (e.g., compromise and negotiation) allows negative emotions (e.g., anger) of children to be calmed in the short term, while in the long term, it can promote mindreading and improve the quality of sibling relationships (Randell and Peterson, 2009).

Therefore, the quality of sibling relationships influences the frequency and quality of sibling interactions; frequent and intense interactions between siblings can shape the development of mindreading abilities and sibling relationships.

### The Dynamic Development of Sibling–ToM Association

It should be noted that the sibling–ToM link is a dynamic process but not a static concept. The reasons are as follows. Firstly, ToM refers to a series of mental states, not just false belief, and children acquire them in a consistent sequence, not limited to preschool age (Wellman, 2017; Peterson and Wellman, 2019). Infants start without ToM, yet by 12 months, babies can understand intentions and goals (Woodward, 1998; Phillips and Wellman, 2005; Olineck and Poulin-Dubois, 2007). During toddlerhood, children gradually understand desires, ignorance, and beliefs, and they attribute individual action to their desires and beliefs in combination (Wellman and Liu, 2004; Wellman et al., 2006). At around the age of 4–5, children show understanding of first-order false belief (Wellman et al., 2001). After then, it takes them 1 or 2 further years to pass second-order false belief tasks (Perner and Wimmer, 1985; Miller, 2009, 2013).

Secondly, the prominent interaction pattern between siblings shifts from complementary interactions to reciprocal interactions as sibling dyads (especially later-born) get older (Howe and Recchia, 2005; Karos et al., 2007; Harrist et al., 2014). Moreover, these two interactions might exert an effect on ToM growth at a different age stage. In the early life of the later-born (aged 1 and under), older siblings might unilaterally promote their ToM by complementary interactions (e.g., teaching, scaffolding). When younger siblings become more effective interactors with age (aged 2 and over), sibling reciprocal interactions (e.g., play, conflicts) allow them to provide one another with fertile context to learn about effective ways to interact with the social world.

Lastly, the nature of sibling mental-state talk is continuously developing with the improvement of children’s ToM and language ability (Tompkins et al., 2018). Before 3 years old, limited by language capabilities, children mainly passively accept mental state knowledge via external inputs (especially parents and elder siblings, Brown et al., 1996). During 3–5 years old, the mental-state terms they can grasp and talk about increase progressively in both frequency and category with ToM growth (Brown et al., 1996; Hughes et al., 2006). Moreover, a recent longitudinal study reveals that children are increasingly able to refer to their own and partner’s internal states simultaneously during play with siblings and friends from early to middle childhood (Leach et al., 2017).

In short, both individual cognitive variables (e.g., ToM and language capabilities) and interaction variables (e.g., quantity and quality of mental-state talks and interaction patterns) are constantly changing with age, which requires researchers to examine the sibling–ToM link from the perspective of development.

## Future Directions

There is some consensus about the sibling–ToM associations that preschoolers with one or more older or child-aged siblings perform better on ToM tasks. The question is far from resolved, however, and may depend upon sibling demographic variables (e.g., birth order, age), sibling interaction variables (e.g., conversations and interaction patterns), environment variables (e.g., cultural background and social disadvantage), as well as the research methods (e.g., specific ToM measures and research designs). By integrating existing literature and unresolved problems, the current review proposes an integrative model for the sibling–ToM association, which underlines the reciprocal effect and dynamic development of the sibling–ToM link. It should be noted that our model is still a theoretical framework, which can generate a series of specific hypotheses. Numerous empirical works need to be done to test these hypotheses, aiming to verify, revise, and expand the model.

### Verification of the Theoretical Model for Sibling–ToM Association

To verify our model, there are two issues worth exploring in future studies. The first is the divergency of developmental trajectories between older and younger sibling–ToM associations. As mentioned above, this “sibling effect” on ToM is reciprocal, and the direction of it can be either negative or positive, but older and younger sibling–ToM associations would show divergent developmental trajectories (see subsection “Summary” for details). Moreover, as children grow older, they may spend less time at home and more time engaging in peer interactions, which may weaken or even eliminate the sibling–ToM association in middle childhood (Calero et al., 2013; Miller, 2013; Lagattuta et al., 2015). To accurately depict divergent developmental trajectories of older and younger sibling–ToM associations, researchers need to conduct more empirical studies with an age-diverse sample and a longitudinal design. The other issue is the dynamic development of the sibling–ToM link. As mentioned in subsection “The Dynamic Development of Sibling–ToM Association,” both individual cognitive capabilities (e.g., ToM and language) and interaction variables (e.g., mental-state talks and interaction patterns) that relate to the sibling–ToM link are dynamic, changing as children grow older, which requires researchers to examine the relationship between siblings and ToM from the perspective of development.

### The Effects of ToM Measures and Research Design

As for the measures of ToM assessment, existing studies on the sibling–ToM association exclusively adopt a variety of explicit, verbal tasks, especially false belief tasks (Hughes and Devine, 2015; Devine and Hughes, 2018). On the one hand, false belief reasoning is only one narrow element of ToM ability and fails to tap into the steps of ToM development (Wellman and Liu, 2004). ToM refers to a series of mental states, including false belief, and children acquire them in a consistent sequence (Wellman, 2017). The “Five-step Developmental Theory of Mind Scale” was established to examine sequences of ToM understanding in 3- to 7-year olds; the order is as follows: diverse desires, diverse beliefs, knowledge access, false belief, and hidden emotion (Wellman and Liu, 2004; Peterson et al., 2005; Peterson and Wellman, 2009). On the other hand, these verbal tasks pose several irrelevant task demands (e.g., general linguistic competence) and thus conceal children’s early capabilities. Because these tasks fail to measure the ToM abilities of children under 3 years old, the existing studies were unable to reveal a sibling–ToM association in the earlier stages of life. During the last two decades, researchers have begun to adopt implicit, non-verbal tasks to assess the mindreading abilities of infants/toddlers (Yott and Poulin-Dubois, 2016). These tasks use infants/toddlers’ spontaneous helping and pointing and eye-gaze patterns (e.g., gaze duration, anticipatory looking) to infer an implicit understanding of ToM (Onishi and Baillargeon, 2005; Yott and Poulin-Dubois, 2012; Slaughter, 2015). With respect to research design, almost all existing studies adopted a cross-sectional design and chose one member of the sibling dyad as the target child. We repeatedly underline the reciprocal effects between siblings on ToM development. However, previous studies focused on one of the sibling dyads that are unable to capture these reciprocal effects. Moreover, our framework lays emphasis on the dynamic development of the sibling–ToM association with age. Existing research using a cross-sectional design fails to capture this constant changing of the sibling–ToM link.

Therefore, to explore the reciprocal effect between siblings across development, future studies should adopt age-appropriate or/and development-sensitive ToM assessment tools, employ a longitudinal design, and gather data from both sibling dyads. Moreover, future works can utilize a cross-lagged panel model to estimate the causal influences on ToM development between siblings over time.

### The Special Impact of Cultural Background

To date, most sibling–ToM association studies have been carried out in the Western context. A few of the cross-cultural studies have found that, unlike Western children, Eastern preschoolers with one or more siblings did not perform better on ToM tasks, and there is no significant association between their ToM performance and the number of older or younger siblings (Shahaeian et al., 2011; Shahaeian, 2015). Shahaeian et al. (2014a, b) have attributed their findings to Eastern and Western cultural differences in parenting practices. Facing sibling conflicts, Iranian parents are more inclined to adopt disciplinary strategies such as “boss” (e.g., deciding for the child, punishing, or controlling), “silence” (e.g., avoidance, silence, or passivity), and “social norms” (e.g., requiring children to abide by social norms) strategies (Shahaeian et al., 2014a, b). These disciplinary strategies may reduce the opportunities for children to become exposed to and participate in mental-state talk among parents, siblings, and target children (Shahaeian et al., 2014a, b).

Similar to Iranian parents, Chinese parents tend to adopt authoritarian parenting strategies (similar to “boss”), which emphasize interpersonal harmony and displaying obedience to authority figures, but discourage expressing personal opinions (Liu et al., 2016; Hou et al., 2019). However, due to the implementation of the “One-Child Policy” in China for more than 30 years, no study has focused on the impact of siblings on the development of Chinese children’s ToM. It is far from clear whether Chinese children who have siblings demonstrate superiority in social understanding like their Western counterparts. With the enactment of the “Universal Two-Child Policy” in 2015, an increasing number of children in China will grow up with siblings. This provides us with a better opportunity to explore the cultural differences between Chinese and Western cultures in the sibling–ToM association. Thus, it is necessary to conduct cross-cultural comparative studies on this issue in the future.

### Concern About the Effects of Social Disadvantage

It is also worth noting that the evidence for the relations between sibling-related factors and ToM appears to be moderated by family background. The sibling–ToM association appears stronger for children from higher-SES families than children from lower-SES families (Hughes and Devine, 2015; Devine and Hughes, 2018). Several studies have consistently found that children from socially disadvantaged families (e.g., low-income or single parents) who have siblings fail to demonstrate superior ToM performance compared to children with no siblings (Cole and Mitchell, 2000; Hughes and Ensor, 2005). Two studies have found that having siblings may even harm the ToM of preschoolers from socially disadvantaged families (Hughes and Ensor, 2005; Tompkins et al., 2013). Contrary to research with middle-income preschoolers, both sibling size and the number of older siblings are negatively correlated with low-income children’s false belief understanding (this negative correlation was regulated by children’s language competence; Tompkins et al., 2013).

Fortunately, this sibling–ToM association of children from low-income families was mediated by the quality of sibling relationships (Hughes and Ensor, 2005). Hughes et al. have suggested that positive sibling relationships could enhance the frequency and quality of sibling interactions, enable siblings to settle conflicts in positive ways (e.g., negotiation), and thus facilitate the reading of minds and emotions. To fully explore whether and how sibling–ToM association is moderated by social disadvantage, two more research avenues are needed: (a) researching the mechanism underlying the effects of social disadvantage on sibling–ToM association and (b) conducting experimental training studies to elucidate the underlying mechanisms of how siblings can enhance children’s understanding of mind and emotion through conversation-based interventions.

### Summary

Together, we hope to clarify the specific effects of older and younger siblings on children’s understanding of minds, the dynamic development of the sibling–ToM association, the similarities and differences of sibling–ToM association under different cultural backgrounds, and the impact of social disadvantage on the sibling–ToM association. All these works would benefit from the verification, revision, and expansion of our reciprocal and dynamic development model for the sibling–ToM association.

## Author Contributions

X-HH wrote the first draft of the manuscript. All the authors contributed to manuscript revision and read and approved the submitted version.

## Conflict of Interest

The authors declare that the research was conducted in the absence of any commercial or financial relationships that could be construed as a potential conflict of interest.
